# Experimental Model for Successful Liver Cell Therapy by Lenti TTR-YapERT2 Transduced Hepatocytes with Tamoxifen Control of Yap Subcellular Location

**DOI:** 10.1038/srep19275

**Published:** 2016-01-14

**Authors:** Mladen Yovchev, Fadi L. Jaber, Zhonglei Lu, Shachi Patel, Joseph Locker, Leslie E. Rogler, John W. Murray, Marius Sudol, Mariana D. Dabeva, Liang Zhu, David A. Shafritz

**Affiliations:** 1 Marion Bessin Liver Research Center, Division of Gastroenterology and Liver Diseases, Albert Einstein College of Medicine, 1300 Morris Park Avenue, Bronx, NY 10461; 2 Department of Pathology, University of Pittsburgh, 200 Lothrop Street, Room S521, Pittsburgh, PA 15261; 3 Department of Anatomy and Structural Biology, Albert Einstein College of Medicine, 1300 Morris Park Avenue, Bronx, NY 10461; 4 Department of Physiology, National University of Singapore, Laboratory of Cancer Signaling & Domainopathies, Yong Loo Li School of Medicine, Block MD9, 2 Medical Drive #04-01, Singapore 117597; 5 Department of Developmental and Molecular Biology, Albert Einstein College of Medicine, 1300 Morris Park Avenue, Bronx, NY 10461.

## Abstract

Liver repopulation by transplanted hepatocytes has not been achieved previously in a normal liver microenvironment. Here we report that adult rat hepatocytes transduced *ex vivo* with a lentivirus expressing a human YapERT2 fusion protein (hYapERT2) under control of the hepatocyte-specific transthyretin (TTR) promoter repopulate normal rat liver in a tamoxifen-dependent manner. Transplanted hepatocytes expand very slowly but progressively to produce 10% repopulation at 6 months, showing clusters of mature hepatocytes that are fully integrated into hepatic parenchyma, with no evidence for dedifferentiation, dysplasia or malignant transformation. Thus, we have developed the first vector designed to regulate the growth control properties of Yap that renders it capable of producing effective cell therapy. The level of liver repopulation achieved has significant translational implications, as it is 2-3x the level required to cure many monogenic disorders of liver function that have no underlying hepatic pathology and is potentially applicable to diseases of other tissues and organs.

The only effective therapy currently available for end stage liver disease is liver transplantation. Because the number of patients that would benefit from a liver transplant far exceeds the number of donor organs available, considerable attention is being focused on potential use of cell transplantation to restore liver function or correct genetic diseases^1^,^2^. Extensive liver repopulation by transplanted hepatocytes has been reported in animal model systems, but this occurs only under highly adverse conditions in which there is massive and continuous liver injury induced in the host by genetic manipulation, as in uPA transgenic mice^3^ or Fah null mice^4^, or host hepatocytes have been subjected to extensive, long-lasting damage, rendering them incapable of cell division, as in retrorsine or monocrotaline treated or x-irradiated rats or mice^5^,^6^,^7^. Under these circumstances, there is a strong selective advantage for proliferation and/or survival of transplanted hepatic cells vs. host cells in the massively injured liver.

In this work, we aimed to identify a gene that will confer on hepatocytes a selective advantage to permit repopulation of the normal liver by these cells. Development of such a system would advance liver cell therapy because a significant number of monogenic-based disorders of the liver that cause major health problems, such as Crigler-Najjar Syndrome, type 1, Ornithine transcarbamylase deficiency and other urea cycle disorders, Familial Hypercholesterolemia, Phenylketonuria and Factor VII deficiency do not have underlying liver injury^8^. Under these circumstances, there is no stimulus for transplanted normal hepatocytes to proliferate in or repopulate the host liver, and in the above genetic disorders, hepatocyte transplantation has been performed with limited, short-term success^2^.

To produce a differentiated hepatic cell that can effectively repopulate the liver in a normal tissue microenvironment, we transduced adult primary rat hepatocytes *in vitro* with a lentivirus containing a human Yap gene to increase their proliferative potential. Yap is the effector gene of the mammalian Hippo kinase signaling cascade, a tumor suppressor pathway that controls organ size with dramatic effects in the liver^9^,^10^,^11^,^12^. Yap is synthesized in the cytoplasm and translocated to the nucleus, where it functions as a transcriptional coactivator of TEADs, p73, Runx2 and other genes (see Fig. S1). Control of Yap function is achieved through phosphorylation by upstream kinases, Mst½ that phosphorylates Lats½, and Lats½ that phosphorylates Yap at amino acid S127, leading to Yap retention in the cytoplasm where it is subsequently degraded^13^,^14^. Yap is a proliferative gene with downstream targets affecting cell growth and the cell cycle (see Fig. S1). Yap also induces expression of 2 anti-apoptotic genes, Birc2 (cIAP1) and Birc5 (survivin). In this study, we have observed that primary rat hepatocytes, transduced with lentivirus hYapERT2 and transplanted into wild type rat liver, repopulate the host liver to a level of 8-10% in six months in a tamoxifen-dependent manner. This level of liver repopulation is sufficient to cure many genetic-based liver disorders, including those in which there is no underlying or ongoing liver injury.

## Results

### Generation of a subcellular location controlled Yap and its functional validation

We previously reported that bipotential rat fetal liver epithelial cells, referred to as fetal liver stem/progenitor cells (FLSPC), repopulate the normal liver after transplantation in conjunction with two-thirds partial hepatectomy (PH)^15^,^16^. After PH, hepatocyte proliferation increases dramatically over 3 days and liver mass returns to normal within 1–2 weeks^17^. However, after liver regeneration is completed, transplanted FLSPC continue to proliferate at a level 4–6 times greater than that observed in adjacent host hepatocytes and replace 20–25% of hepatocyte mass within 6 months^16^. Despite these remarkable findings, it is unlikely that FLSPC will be used clinically for liver cell therapy because of the high number of fetal liver cells required for repopulation, the probable need for cells pooled from multiple donors and ethical concerns regarding acquisition of fetal liver cells for human use.

We hypothesized that transduction of primary rat hepatocytes with a gene that will increase their proliferative activity and express other properties that foster liver repopulation will enable the transduced cells to effectively repopulate a normal or near normal liver. Since Yap is a proliferative gene and effector of the mammalian Hippo signaling pathway, we queried our published mouse microarray data^18^ for expression of core Hippo signaling genes in FLSPC vs. adult liver (Fig. S2A). Mst½ expression was decreased ~3.0-fold in FLSPC vs. adult liver, Lats½ was unchanged and Yap expression was increased ~2.0 fold. Birc5 (survivin), a downstream target of Yap^9^, was increased 20 fold in mouse FLSPC vs. adult liver. In addition, Yap was the only major anti-apoptotic gene hyperexpressed in rat FLSPC compared to adult hepatocytes (Fig. S2B).

Based on these findings, we selected Yap as a candidate gene for inducing liver repopulation by lentivirus transduced hepatocytes in a physiologically normal liver. We designed a system for tightly controlled, low to moderate expression of Yap, because transgenic mice hyperexpressing Yap under control of the tet operon^9^,^10^, as well as Mst½ null mice that express high levels of endogenous Yap^19^,^20^,^21^, develop massive liver enlargement (hyperplasia) leading to hepatocellular carcinoma^9^,^10^,^19^,^20^,^21^,^22^. Human HCCs and cholangiocarcinomas, as well as many other human tumors, also hyperexpress Yap^11^,^23^,^24^.

For current studies, we used hYap1-2β isoform, a human Yap1 cDNA containing two WW domains and also with two tandem 5′ flag sequences for detection by immunohistochemistry^25^. To turn Yap activity on and off in transplanted, transduced hepatocytes, we fused it to the estrogen receptor (ER) to control its cytoplasmic-to-nuclear transfer by tamoxifen administration. For these studies, we used ERT2, a modified estrogen receptor to which 4-OH-tamoxifen (the active metabolite of tamoxifen) binds with very high affinity, whereas 17β-estradiol (the active estrogenic hormone in mammals) exhibits negligible binding^26^. For *in vitro* studies, Yap was driven by the ubiquitous EF1 promoter and for *in vivo* studies, Yap was placed under control of the hepatocyte-specific transthyretin (TTR) promoter (Fig. S3).

**Figure 1:**
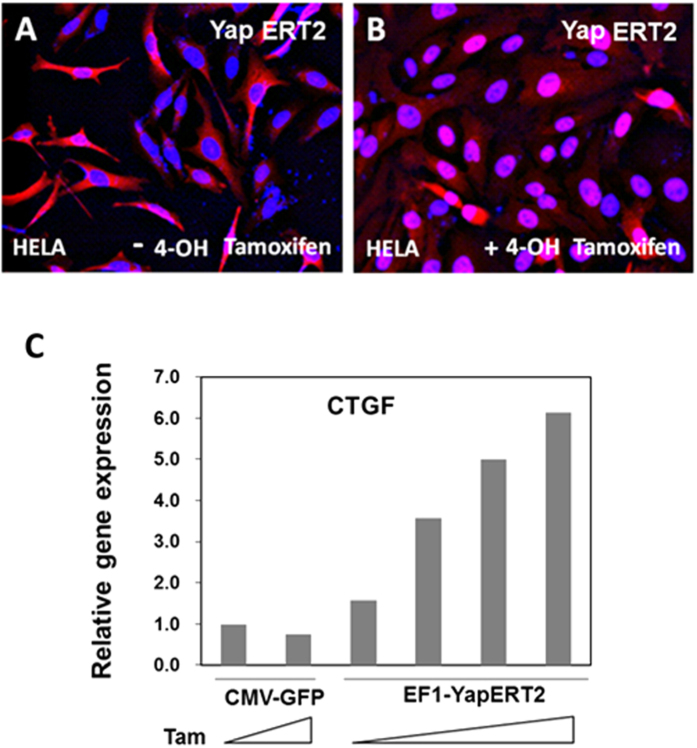
*In vitro* validation of lenti EF1-hYapERT2 expression and function. HeLa cells or NIH3T3 cells were transduced overnight in culture with lenti EF1-hYapERT2 and maintained in culture for 3-4 days. Immunohistochemical detection of hYapERT2 (containing two flag sequences at the 5’ end of Yap) in HeLa cells maintained in the absence (**A**) or presence (**B**) of 100 nM 4-OH tamoxifen. (**C**) Tamoxifen dose dependent expression of CTGF mRNA quantified by qRT PCR in RNA extracts from densely cultured NIH3T3 cells transduced with lenti EF1-hYapERT2 and maintained in culture for 3 days. Expression of CTGF mRNA in NIH3T3 cells transduced with lenti CMV-GFP in the absence of 4 OH-tamoxifen addition is used as a control and is set at 1.0. In C, the range in 4 OH-tamoxifen addition to the cell culture medium is from 0 to 400 nM.

In HeLa cells transduced with lenti EF1-hYapERT2 and maintained in culture for 4 days, nearly all of the Yap expressed from the hYapERT2 vector was cytoplasmic in the absence of 4-OH tamoxifen (Fig. 1A). In the presence of 4-OH tamoxifen, most of the hYapERT2 was located in the nucleus (Fig. 1B). To demonstrate function of lenti EF1-hYapERT2, we tested virally transduced NIH3T3 cells for expression of CTGF mRNA, a major downstream target of Yap^21^,^27^ in the presence or absence of 4-OH tamoxifen. Using densely cultured lenti hYapERT2 transduced NIH3T3 cells, there was a progressive increase in CTGF mRNA expression with increasing concentration of 4-OH tamoxifen in the medium, with 6-fold greater CTGF expression at 400 nM 4-OH tamoxifen compared to expression in the absence of 4-OH tamoxifen (Fig. 1C). These results demonstrate that Yap expressed from this vector depends on tamoxifen for nuclear localization and transactivating activity.

### Repopulation of rat liver by lentiTTR-hYapERT2 transduced hepatocytes

**Figure 2:**
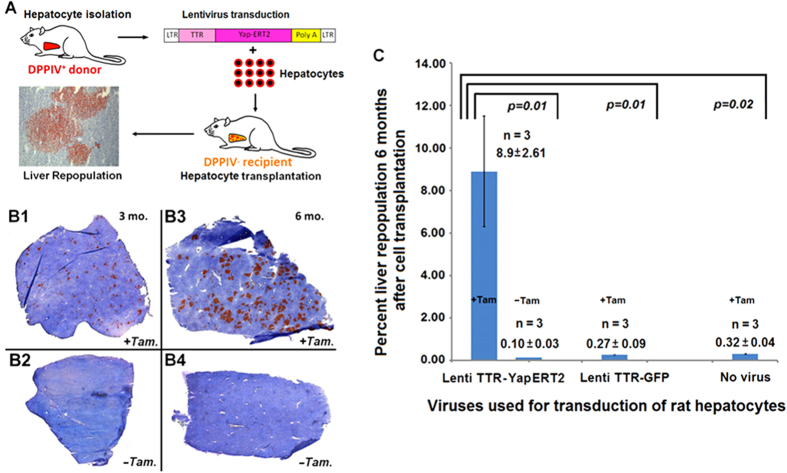
Liver repopulation by lenti TTR-hYapERT2 transduced hepatocytes transplanted into normal adult rat liver. (**A**) Hepatocyte transplantation protocol. (**B**) Detection of lentivirus TTR-hYapERT2 transduced hepatocytes in DPPIV^−^ host liver by DPPIV enzyme histochemistry. Clusters of DPPIV^+^ cells are clearly visible at 3 mo. after cell transplantation in tamoxifen fed rats (B1). In the absence of tamoxifen feeding, clusters of transplanted cells are not apparent (B2), but individual cells and small groups of cells can be seen at higher magnification (not shown). At 6 mo. after transplantation of lenti TTR-hYapERT2 transduced hepatocytes, clusters of DPPIV^+^ cells are much larger in tamoxifen fed rats, and in some instances comprise whole liver lobules (B3). In the absence of tamoxifen feeding, transplanted DPPIV^+^ cells are still not visible at low magnification (B4). (**C**) Quantification of liver repopulation by scanning digital images, using a Zeiss Axio Observer Z1 microscope and image J software, was performed in DPPIV^−^ rats transplanted with lenti TTR-hYap ERT2 transduced hepatocytes, lenti TTR-GFP transduced hepatocytes or non-transduced hepatocytes, all maintained on tamoxifen feeding (+Tam) or for rats transplanted with lenti TTR-hYapERT2 transduced hepatocytes maintained on normal chow (−Tam).

To track the fate of transplanted hepatic cells and monitor liver repopulation, we used an inbred Fischer (F) 344 rat model system based on detection of a cell surface marker gene, dipeptidylpeptidase IV (DPPIV)^28^ that we and others have used extensively^5^,^7^,^15^,^16^,^29^,^30^,^31^. Hepatocytes isolated from wild-type (WT) DPPIV^+^ F344 rats were transduced in suspension with lentivirus TTR-hYapERT2 (see Supplementary Methods for lentivirus transgene vector production and virus preparation) and transplanted into the liver of syngeneic DPPIV^−^ rats that exhibit an otherwise normal liver phenotype (Fig. 2A). During the first two weeks, isolated single DPPIV^+^ cells or groups of 2-3 DPPIV^+^ cells were identified throughout the hepatic parenchyma. The size of these cell collections increased progressively over time and, during continuous tamoxifen feeding for 3 months, the size of DPPIV^+^ clusters increased to a maximum of 75-100 cells/cluster in two-dimensional sections, leading to ~3.0% liver repopulation (Fig. 2B1). In the absence of tamoxifen feeding, isolated DPPIV^+^ cells and small collections of up to 5-10 DPPIV^+^ cells were noted throughout the liver, but there was no significant repopulation (Fig. 2B2). The size of DPPIV^+^ clusters increased significantly between 3 and 6 months in the presence of tamoxifen feeding to 100-200 cells/cluster (Fig. 2B3), but there was still no significant liver repopulation at 6 months in the absence of tamoxifen feeding (Fig. 2B4). Quantification of liver repopulation by lenti TTR- hYapERT2 transduced hepatocytes at 6 months after cell transplantation was 8.9 ± 2.61% (SEM) in the presence of tamoxifen, but was 0.10 ± 0.03% (SEM) in the absence of tamoxifen (P = 0.01) (Fig. 2C). Under tamoxifen feeding, liver repopulation by transplanted lentiTTR-GFP transduced hepatocytes was 0.27 ± 0.09% (SEM) (P = 0.01 compared to lenti TTR-hYapERT2) and 0.32 ± 0.04% (SEM) with non-transduced hepatocytes (P = 0.02 compared to lenti TTR-hYapERT2). These P values were determined by a 2-tailed Student’s t test. When we used the non-parametric Wilcoxon Rank-sum test (which is more stringent when sample sizes are small), the P values were 0.04 for each of the same three experimental pairs.

**Figure 3:**
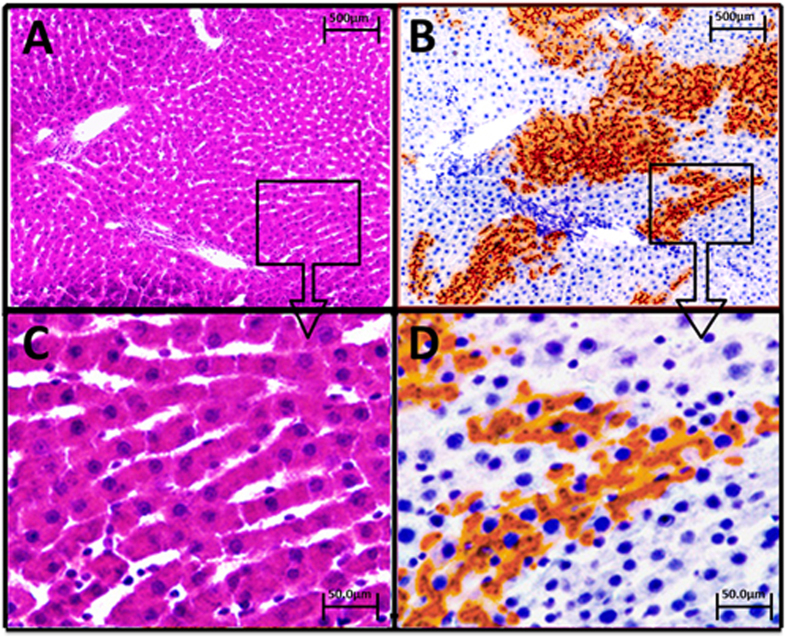
Incorporation of transplanted, lenti TTR-hYapERT2 transduced hepatocytes into liver parenchymal plates. Animals sacrificed 6 mo. after hepatocyte transplantation and maintained on tamoxifen diet. (**A,C)** H&E staining of a representative section of repopulated rat liver at orig. mag. 4× and 60×, respectively. (**B,D**) serial section of the same liver region stained for DPPIV at orig. mag. 4× and 60×, respectively. On both H & E and DPPIV staining, liver tissue post lenti TTR-YapERT2 transduced hepatocyte transplantation was totally normal and transplanted cells and their progeny were incorporated into a totally normal liver structure, without evidence for dedifferentiation, dysplasia or malignant transformation.

**Figure 4:**
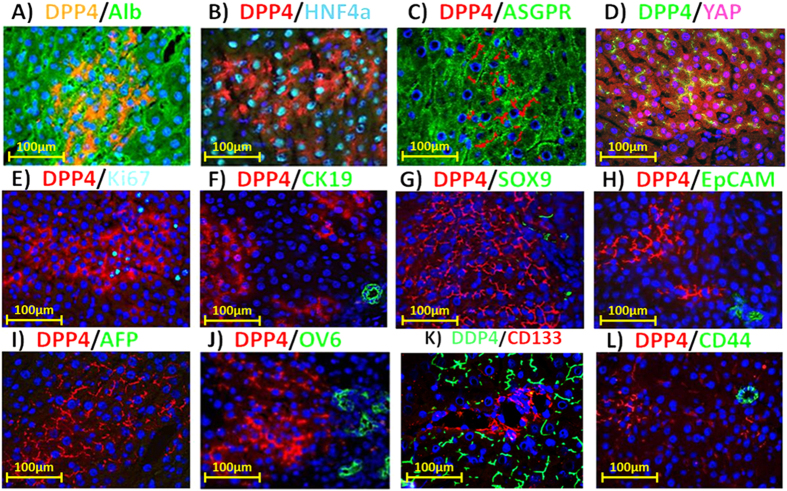
Double label fluorescent immunohistochemistry for DPPIV and selected marker genes in repopulating clusters at 6 mo. after transplantation of lenti TTR-hYapERT2 transduced hepatocytes. The specific protein paired with DPPIV and the fluorescent color generated by the secondary antibody for each protein are indicated in panels A–L. Examples of genes related to specific hepatocytic function that were expressed in DPPIV^+^ cells at a level comparable to surrounding host liver include albumin, HNF4α and ASGPR (**A–C**). Yap expression was increased in both the nucleus and cytoplasm of repopulating DPPIV^+^ cells compared to surrounding liver (**D**), Ki67 was increased by 25–30% compared to surrounding host liver (**E**), progenitor/biliary epithelial cell markers, CK19, Sox9 and EpCAM, were not expressed in DPPIV^+^ cells (**F–H**), indicating that repopulating TTR-hYapERT2 transduced hepatocytes retained a differentiated hepatocytic phenotype. Other markers indicative of an undifferentiated, progenitor or tumorigenic phenotype in repopulating DPPIV^+^ cells, including AFP, OV6, CD133 and CD44, were also negative (**I–L)**, respectively). Nuclei stained with Dapi (blue). Orig. mag. = 60X.

Hematoxylin and eosin (H&E) staining and DPPIV enzyme histochemistry on serial sections of frozen liver tissue from DPPIV^−^ F344 rats transplanted with lenti TTR-hYapERT2 transduced DPPIV^+^ hepatocytes under continuous tamoxifen feeding showed no detectable abnormality in the liver lobular structure (Fig. 3). Although transplanted DPPIV^+^ hepatocytes expanded approximately 50–100-fold during the 6 month repopulation process, they formed normal one cell thick hepatic plates that were totally integrated into the host hepatic parenchyma without compressing or distorting surrounding liver tissue (Fig. 3A,B). At 60X magnification, transplanted hepatocytes were morphologically indistinguishable from host hepatocytes, except for expression of DPPIV (Fig. 3C,D). There was no evidence for atypia, dysplasia or malignant transformation of hepatocytes (transplanted or endogenous) in repopulated host liver. Double-label immunohistochemistry for DPPIV and several genes related to specific hepatocytic function showed that albumin, HNF4α and ASGPR were expressed in virtually all DPPIV^+^ cells at levels comparable to surrounding host hepatocytes (Fig. 4A–C). Human Yap was expressed in both the nucleus and cytoplasm in 95% of DPPIV^+^ cells (Fig. 4D), of which 79% were positive for Yap in both the nucleus and cytoplasm, 18% were positive for Yap only in the cytoplasm and 3% were positive for Yap only in the nucleus. Four hundred and thirty seven DPPIV^+^ cells contained in 6 repopulation clusters in this tissue specimen were evaluated to obtain these results. Ki67 expression was increased by 25–30% in DPPIV^+^ cells (Fig. 4E). None of the DPPIV^+^ cells expressed progenitor/biliary epithelial cell markers (CK19, Sox9 and EpCAM) (Fig. 4F–H). Furthermore, DPPIV^+^ cells did not express AFP, OV6, CD133 and CD44, markers indicative of an undifferentiated hepatic progenitor, biliary or tumorigenic phenotype (Fig. 4I–L).

### Aberrant repopulation of rat liver by lenti TTR-hYapS127A transduced hepatocytes

**Figure 5:**
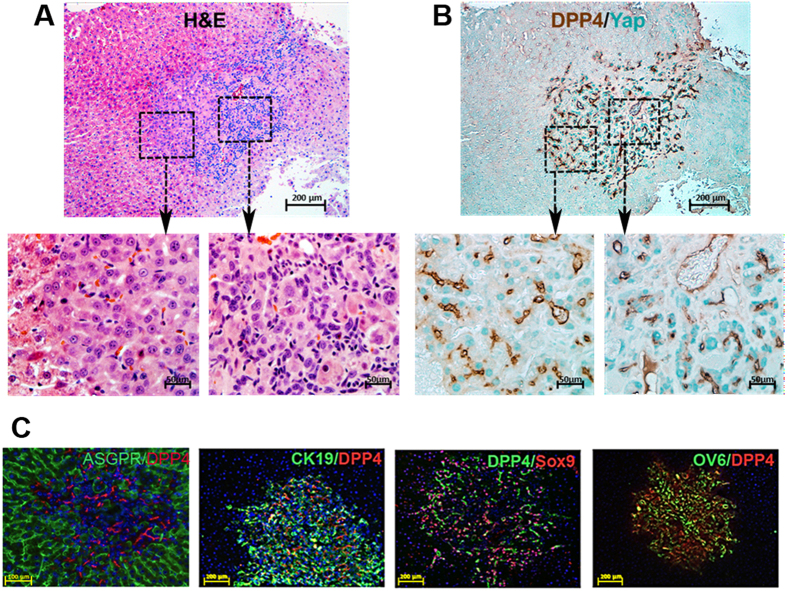
Liver repopulation by hepatocytes transduced with lentivirus TTR-hYapS127A. Rats were maintained on a normal chow diet and sacrificed 5 months after hepatocyte transplantation. **(A)** H&E staining showing an area of the liver containing a repopulation cluster of transplanted hYapS127A-expressing hepatocytes. Two selected areas, as marked, were photographed at 60X and shown as “left” or “right”. **(B**) Double-label immunohistochemistry for DPPIV (brown) and Yap (green) in a serial section with enlarged (60X) photographs as in **(A)**. **(C)** Expression of Yap and hepatocytic function marker ASPGR; or progenitor/biliary markers CK19, Sox9, and OV6. ASGPR/DPP4 (orig. mag., 20X); CK19/DPP4, DPPIV/Sox9 and OV6/DPPIV (orig. mag., 10X).

Camargo *et al*.^10^ developed a Yap transgenic mouse model in which the Yap gene contains a mutation in serine 127 to alanine (YapS127A), so that Yap protein is not phosphorylated at this critical amino acid and is retained in the nucleus, where it exhibits constitutive Yap function. Using lineage tracing technology, Yimlamai *et al*.^27^ have recently demonstrated in this model that within a few days after induction of Yap expression by treating the mice with AAV Cre recombinase and Dox administration, individual hepatocytes proliferate and dedifferentiate into bipotential hepatic epithelial progenitor cells and more differentiated biliary epithelial progenitor cells. We, therefore, transplanted DPPIV^+^ rat hepatocytes transduced with lenti TTR-YapS127A into DPPIV^-^ recipients under the identical conditions as used for DPPIV^+^ lenti TTR-YapERT2 hepatocytes, except that tamoxifen was not administered. There was a similar level of repopulation of the host liver at 5 months; however, on H & E staining (Fig. 5A), many of the cells in the repopulation clusters, confirmed by double-label immunohistochemistry for DPPIV/Yap in serial sections (Fig. 5B), were smaller than mature hepatocytes and a normal liver parenchymal cord structure was not developed in these areas (Fig. 5A, also see enlarged image at left). In the same cluster, pleomorphic cells with nuclei of varying sizes and shapes can be seen arranged in pseudoductular structures (Fig. 5A and enlarged image at right). Strikingly, YapS127A expression is mostly restricted to the nuclei of cells with an hepatocyte-like morphology (Fig. 5B and enlarged image at left) but accumulated mostly in the cytoplasm of cells in the pseudoductular area (Fig. 5B and enlarged image at right). This latter finding suggests a functional cytoplasmic role of this particular Yap mutant in promoting a biliary/ductal phenotype.

On double label immunohistochemistry for DPPIV and ASGPR (a marker for mature hepatocytes) or CK-19, Sox9 or OV6 (markers for bipotential hepatic epithelial progenitor cells, biliary epithelial progenitor cells or mature biliary epithelial cells) (Fig. 5C), less than half of the DPPIV^+^ cells were positive for ASGPR (Fig. 5C), but the majority were positive for CK-19, Sox9 or OV6 (Fig. 5C). These results are consistent with those reported by Yimlamai *et al*.^27^ for the phenotypes of repopulating cells in YapS127A transgenic mice after AAV cre recombinase treatment and Dox administration. Therefore, YapS127A expressing hepatocytes in both mice and rats exhibit substantial plasticity in which proliferation is accompanied by dedifferentiation and phenotypic conversion of hepatocytes to a more primitive ductal/progenitor phenotype. In contrast, YapERT2 promoted hepatocyte proliferation is not accompanied by this phenotypic conversion.

## Discussion

Based on studies conducted over many decades and confirmed by recent lineage tracing studies, it has been concluded that after acute liver injury or loss of hepatic mass through partial hepatectomy, the liver regenerates by proliferation of preexisting mature hepatocytes and stem or progenitor cells do not contribute significantly to this process^32^,^33^,^34^. Other studies have reported that cells with the morphologic appearance and gene expression characteristics of biphenotypic hepatic epithelial progenitor cells, proliferate and form atypical duct-like structures in rodent models of chronic liver injury^35^,^36^,^37^, as well as in human chronic liver disease^38^,^39^. Some of these cells have been reported to differentiate into hepatocytes but their role, as well as that of liver stem cells, in normal liver physiology or hepatocyte replacement during liver regeneration remains controversial^40^,^41^,^42^. Other studies have reported that stem or progenitor cells of non-epithelial lineage origin (e.g., hematopoietic or other mesenchymal progenitor cells, stellate cells or fibroblasts) can differentiate into hepatocytes^43^. Biliary epithelial cells can also lineage-convert into hepatocytes^44^ and hepatocytes can differentiate into biliary duct cells under conditions of severe biliary injury^30^. However, most recent lineage fate tracing studies in the mouse have reported that the contribution of stellate cells and biliary epithelial cells or biliary epithelial progenitor cells to hepatocyte repopulation is negligible, even in liver injury/disease models^34^,^45^,^46^,^47^.

In a normal liver microenvironment, transplanted hepatocytes from a normal donor engraft^48^,^49^ but do not significantly repopulate the host liver^29^, because they do not have a selective advantage over host hepatocytes. In contrast, hepatocytes isolated from Kip1 cyclin kinase inhibitor (p27^Kip1^) null mice that show increased cell cycling and multiorgan hyperplasia, including the liver^50^, produce modest repopulation in recipient mice (~3.27%)^51^. However, this occurred only when the host was subjected to repeated cycles of liver injury/regeneration induced by 8 weekly IP injections of CCl_4_^51^.

Using hYapERT2 transduced hepatocytes, we have repopulated a normal host liver to a level higher than that achieved with p27^Kip1^ null hepatocytes, without the need for continuous or repeated liver injury^51^. Yimlamai *et al*.^27^ have reported that turning on Yap expression in hepatocytes in Yap S127A transgenic mice leads to their proliferation and dedifferentiation into biphenotypic epithelial progenitor cells. The Yap vector used in the present study contained a wild-type human Yap1 coding sequence under control of the much weaker TTR-promoter and was linked to the estrogen receptor to regulate Yap function by tamoxifen administration. Using hepatocytes transduced with lenti TTR-YapERT2 and transplanted into the context of a normal liver microenvironment, we did not observe morphologic abnormalities or expression of a biliary epithelial progenitor phenotype by DPPIV^+^ cells in the recipient liver. Interestingly, when we transplanted rat hepatocytes transduced with a lentivirus expressing a TTR-hYapS127A gene into normal adult rat liver under the same conditions as used for TTR-YapERT2 (but in the absence of tamoxifen), we observed results consistent with those reported by Yimlamai *et al*.^27^ Our contrasting results with YapERT2 vs. YapS127A transduced hepatocytes transplanted into a normal liver microenvironment indicates that the differences in plasticity or phenotypic behavior of repopulating hepatic cells transduced with these vectors are due to differences in the innate properties of these two Yap constructs. The unexpected finding of high accumulation of Yap S127A in the cytoplasm of repopulating DPPIV^+^ cells that exhibit a dedifferentiated progenitor or biliary-like phenotype suggests that reduced clearance of this protein may lead to activation or complex formation with β-catenin, as observed by Tao *et al*.^52^ in hepatoblastomas and cholangiocarcinomas, and upregulation of Jagged-1 and activation of Notch signaling, as reported by Tschaharganeh *et al*.^53^ in HCC.

Concerning the potential impact of our study, it is generally agreed that the amount of cell replacement required to achieve effective therapy in inherited metabolic disorders of the liver, such as in Crigler-Najjar Syndrome, Type 1 (CN1), Familial Hypercholesterolemia, Factor IX deficiency, or ornithine transcarbamylase deficiency, etc.) is 3–5%^54^ and several studies have reported a partial therapeutic response when hepatocytes comprising 1–2% of total hepatocytic mass were transplanted into patients with CN1^55^,^56^. However, the therapeutic response in these and other studies^2^ was only temporary, most likely because the transplanted cells did not proliferate after their transplantation and were lost over time. We have overcome low repopulation and loss of transplanted hepatocytes by engineering donor cells to express the proliferative gene hYap fused to ERT2 with function controlled by tamoxifen. The level of hepatocyte replacement achieved in our DPPIV model transplantation system is ~2-3X that necessary to cure many monogenic-based liver diseases. Moreover, since lentivirus transgene integration into the host genome is stable, proliferation of transplanted lenti-TTR-hYap ERT2 transduced hepatocytes can potentially be reactivated periodically by tamoxifen administration to boost cell replacement and maintain effective therapy. We, therefore, anticipate the possibility of achieving life-long therapy with the methods we have developed. Since the transduced hepatocytes with augmented proliferative potential we used here are otherwise normal, human hepatocytes transduced with this vector may be universally applicable for treatment of patients with a wide variety of liver diseases, with further potential for broader application to diseases of other tissues and organs.

## Methods

### Animals

Inbred male DPP4^+^ F344 rats (Taconic Farms, German Town, NY) were used as hepatocyte donors. Syngeneic mutant DPP4^−^ F344 rats (cell recipients) were provided by the Special Animal Core, Marion Bessin Liver Research Center, Albert Einstein College of Medicine. All procedures were humane and approved by the Einstein Animal Care Use Committee. The methods were carried out in accordance with the approved guidelines. Rats were fed a diet containing 500 mg/kg tamoxifen citrate prepared by Bio-Serve, Frenchtown, NJ or a normal rodent chow diet, as indicated. Animals receiving either short-term (2 weeks) or long-term (6 months) tamoxifen feeding did not show elevation of ALT values or histologic evidence of chronic liver injury.

### Hepatocyte isolation

Rat livers were perfused with 5 mM EGTA solution, followed by Liberase Blenzyme solution (7 U/100 mL; Roche Applied Science, Indianapolis, IN). After perfusion, livers were excised and minced in DMEM/10% FBS. The cells suspension was filtered through an 80-μm nylon mesh and centrifuged for 2 minutes at 50 × g at room temperature (RT). The pellet was washed 3 times, resuspended in DMEM/10%FBS and mixed with an equal volume of Percoll (GE Healthcare Bio-Sciences Corp, Piscataway, NJ) solution (containing Percoll/10 × HBSS, 9:1) and centrifuged for 10 minutes at 50 × g at 4 °C. The cell pellet was washed 2 times at 200 × g, resuspended in DMEM/10% FBS and used for *ex vivo* transduction with lentiviruses and cell transplantation.

### Lentiviral vector and virus production

Preparation of lentivirus vectors and virus production are detailed in Supplementary Methods.

### Transduction of cultured HeLa cells or NIH 3T3 with lentiviruses

3 × 10^5^ HeLa cells or NIH 3T3 cells were plated on 3.5 cm culture dishes in DMEM/5% FBS. The next morning, cells were infected with lentivirus particles containing EF1-YapERT2 at a concentration of 500 VP/cell. Medium was changed after 24 h and after 2–4 days, cells were fixed with 4% PFA at room temperature for 20 min. or collected in TRIzol reagent for RNA extraction. A 10^−2^ M stock solution of 4-OH tamoxifen in methanol was stored at −20 °C. To prepare a working solution, 4-OH tamoxifen was diluted to 1 × 10^−7^ M (0.1 μM) in Blocks medium and applied to cultured cells for 2-4 days.

### Transduction of primary rat hepatocytes with lentiviruses

Primary rat hepatocytes were transduced in suspension by a modification of the procedure of Nguyen, *et al*.^57^ 5 × 10^6^ freshly isolated DPPIV^+^ hepatocytes were washed 2 times with Block’s medium at 100 × g in a 50 ml tube and the resuspended cells were mixed with 500 VP/cell lentiviruses in 500 μl Block’s medium containing 5% FBS, 25 ng/ml EGF and 2 × ITS. The cells were incubated at room temperature for 4 h with gentle agitation. The cells were washed 2 times and spun down at 100 × g in 40 ml of fresh DMEM/10% FBS and the final pellet was resuspended in DMEM/10% FBS to a concentration of 1.0 × 10^7^ cells/ml. Before transplantation, virally transduced hepatocytes were checked for viability by trypan blue dye exclusion and cells were transplanted when their viability was 80% or higher.

### RNA Isolation, Reverse-Transcriptase Polymerase Chain Reaction (RT-PCR) and Quantitative, Real Time PCR

RNA was isolated from cultured cells using Trizol (Life Technologies, Carlsbad, CA) according to the manufacturer’s instructions and resuspended in RNase free water. RNA was further treated with DNAse I (NEB, Ipswich, MA) for 30 min and purified using the RNeasy Mini Kit (Qiagen, Germany). All reverse transcriptase reactions were carried out with the Verso cDNA Synthesis Kit (Thermo Scientific, Waltham, MA), according to the manufacturer’s protocol. Rat specific primers for different genes with annealing temperature of 60 °C for all were chosen with the Primer3 program and are listed in Table S1. The expression level of glyceraldehyde-3-phosphate dehydrogenase (GAPDH) was used as an internal control. For Quantitative, real time PCR, 20 ng cDNA were mixed with 1× Choice Taq Blue Mastermix (Denville Scientific Inc, Metuchen, NJ) and 0.5 mM primers and amplified 23 to 33 cycles. Real time PCR was performed in triplicate for each gene. Each SYBR Green assay was performed in a 12 μl total reaction volume that included 6 μl of 2× SYBR Green Power master mix (Applied Biosystems, Foster City, CA), 250 nM of each primer and 20 ng of template cDNA. Assays were run on a 7500FAST instrument (ABI) under standard conditions recommended by the manufacturer and were: 95 °C for 10 min, followed by 40 cycles of 95 °C for 15 sec, and 60 °C for 1 min followed by melting curve analysis. Data were analyzed using 7500 ABI software, V2.0.6. Fold difference in gene expression was determined by the ∆∆Ct method.

### Quantification of liver repopulation by transplanted cells

5 μm frozen liver sections stained for DPPIV expression were imaged using a Zeiss AxioObserver microscope with the 5×, 0.16 NA objective and images were captured with a Zeiss Axiocam HRc color camera. Sequential images were mapped and stitched together using Zeiss Axiovision software (version 4.8). Total area and DPPIV positive area of liver sections were quantified using ImageJ software (Rasband, W.S., ImageJ, U.S. National Institutes of Health, Bethesda, Maryland, USA, http://imagej.nih.gov/ij/, 1997–2014).

### Immunofluorescence microscopy

For detection of cytoplasmic proteins, tissue sections were permeabilized with 0.3% Triton X-100. Blocking was with 5% normal serum from the animal species in which the secondary antibody was raised and 2% bovine serum albumin (BSA). Primary antibodies were applied overnight at 4 °C in 2% normal serum/2% BSA. The secondary, fluorescent conjugated antibody was applied for 40 min at room temperature. Sections were counterstained with 4′, 6-diamidino-2-phenylindole (DAPI). Fluorescence images were obtained with a Nikon Eclipse TE 2000-S fluorescence microscope. Primary and secondary antibodies used in the different IF analyses are given in Table S2.

### Statistics

Data are shown as mean ± SEM. Statistical significance was determined by a 2-tailed Student’s *t* test.

## Additional Information

**How to cite this article**: Yovchev, M. *et al*. Experimental Model for Successful Liver Cell Therapy by Lenti TTR-YapERT2 Transduced Hepatocytes with Tamoxifen Control of Yap Subcellular Location. *Sci. Rep.* **6**, 19275; doi: 10.1038/srep19275 (2016).

## Supplementary Material

Supplementary Information
