# Supplementary material for: Experimental Model for Successful Liver Cell Therapy by Lenti TTR-YapERT2 Transduced Hepatocytes with Tamoxifen Control of Yap Subcellular Location
Source: Sci Rep. 2016 Jan 14;6:19275. doi: 10.1038/srep19275 (PMC4725878; doi:10.1038/srep19275)
Supplement: Supplementary Information [file srep19275-s1.doc]

**SUPPLEMENTARY INFORMATION**

**Experimental Model for Successful Liver Cell Therapy by Lenti TTR-YapERT2 Transduced Hepatocytes with Tamoxifen Control of Yap Subcellular Location**

Mladen Yovchev^a^, Fadi L. Jaber^a^, Zhonglei Lu^a,e^, Shachi Patel^a^, Joseph Locker^b^, Leslie E. Rogler^a^,

John W. Murray^c^, Marius Sudol^d^, Mariana D. Dabeva^a^, Liang Zhu^a,e^ and *David A. Shafritz^a^

**
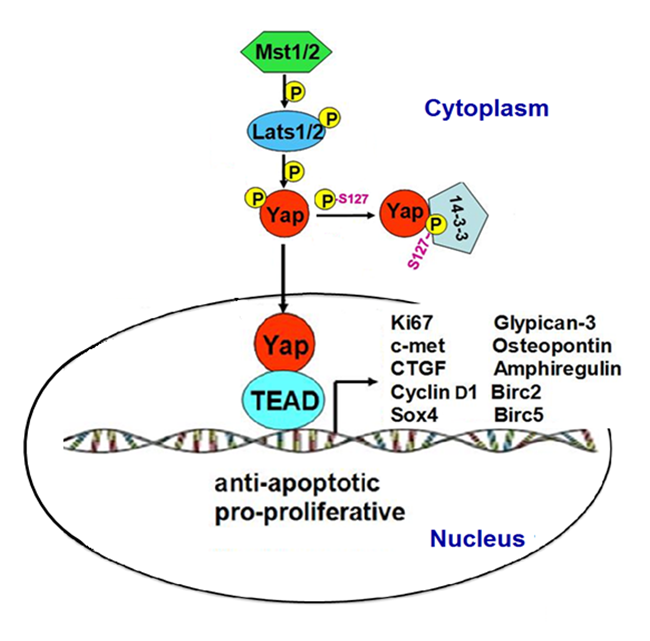
**

**
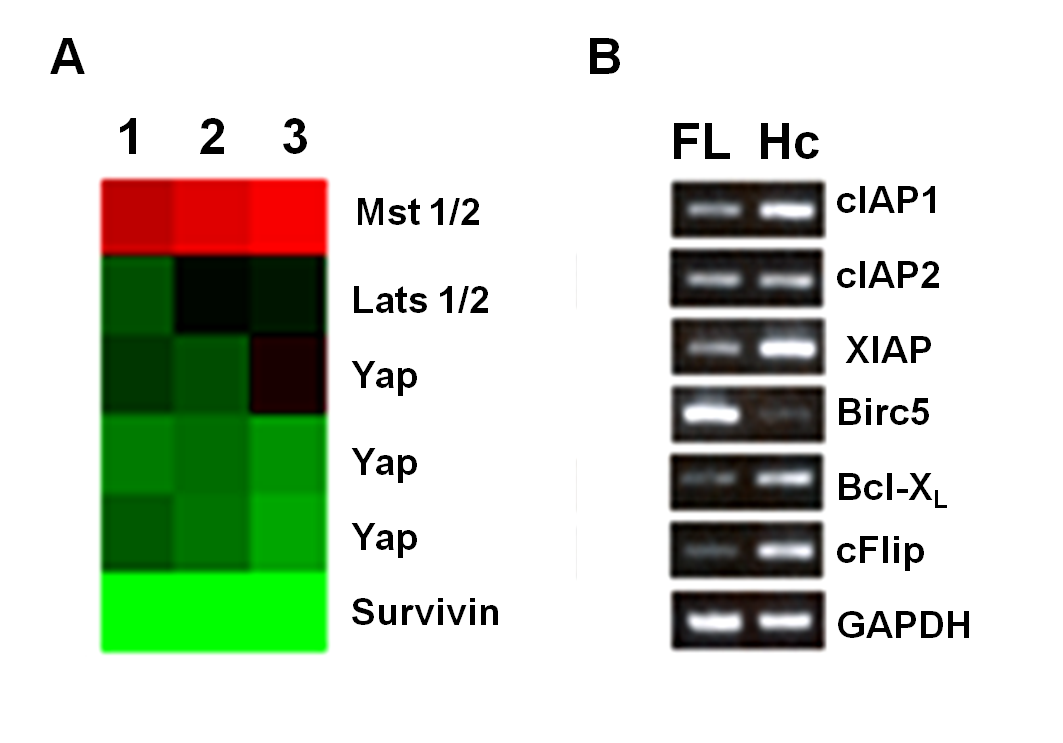
**

**Supplementary Fig. 1.** **Core components and essential steps in mammalian Hippo signaling.**

**Supplementary Fig. 2. Expression of core Hippo signaling genes in FLSPC vs. adult liver. A)** Heat maps from microarray analysis indicating the relative ratio for expression of mst½, lats½, Yap and survivin in purified mouse FLSPC vs. adult mouse liver, taken from published microarray data, Ref. 18 (Nierhoff et al., 2007). Mst½, decreased 3.0 fold; lats½, unchanged; Yap, increased 2.0 fold; survivin, increased 20 fold. For Yap, 3 different ESTs were present on the microarray chips. Lanes 1, 2 and 3 represent data from sample pairs from 3 separate experiments. **B)** RT-PCR for mRNA expression of anti-apoptotic genes in purified rat FLSPC (FL) vs. adult hepatocytes (Hc), using GAPDH as loading control. Anti-apoptotic genes Bcl-2 and mcl are not expressed in rats and were not included in this analysis.

**Immunofluorescence microscopy**

For detection of cytoplasmic proteins, tissue sections were permeabilized with 0.3% Triton X-100. Blocking was with 5% normal serum from the animal species in which the secondary antibody was raised and 2% bovine serum albumin (BSA). Primary antibodies were applied overnight at 4°C in 2% normal serum/2% BSA. The secondary, fluorescent conjugated antibody was applied for 40 min at room temperature. Sections were counterstained with 4', 6-diamidino-2-phenylindole (DAPI). Fluorescence images were obtained with a Nikon Eclipse TE 2000-S fluorescence microscope. Primary and secondary antibodies used in the different IF analyses are given in Table S2.

**
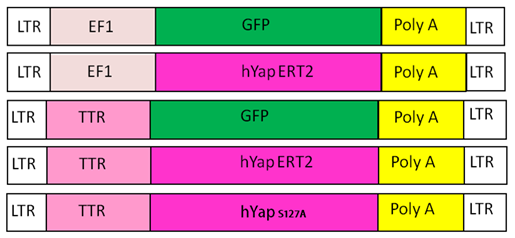
**

**Supplementary Fig. 3. Lentivirus Transgene Constructs Used in these Studies.**

**SUPPLEMENTARY METHODS**

**Plasmid *pCCLsin.cPPT.hEF1.GFP.WPRE*.**

To create a lentivirus transfer vector carrying the GFP gene under control of the EF1 promoter, two different parental plasmids were used: *pCCLsin.cPPT.hPGK.GFP.WPRE* (providing the vector backbone) and pEF1_GFP (containing the EF1 promoter and GFP sequence in forward orientation). The *pCCLsin.cPPT.hPGK.GFP.WPRE* plasmid contains unique restriction sites flanking the PGK promoter/GFP fragment; EcoRV at the 5’ end and SalI at the 3’ end. After double digestion, two fragments are produced: vector (6552bp) and PGK/GFP (1275bp). The vector (6552bp) was isolated from an agarose gel, blunt-ended and column purified. Because of a lack of matching unique restriction sites in both plasmids, the EF1/GFP sequence was amplified with primers for EF1 and GFP. The vector and EF1/GFP containing fragments were ligated to generate the plasmid *pCCLsin.cPPT.hEF1.GFP.WPRE* (Supplementary Fig. 3).

**Plasmid *pCCLsin.cPPT.hTTR.noORF.WPRE*.**

The TTR promoter was PCR-amplified from plasmid pRRL_TTR_GFP_240-1, using primer pairs TTR-XhoI-F and TTR-R. The amplified fragment (564bp) was cloned into the SmaI site of pBluescript to generate plasmid pBS-TTR. The primers were designed to contain a unique restriction site to allow easier promoter transfer into different vectors. Plasmid pBS-TTR was used to create a transfer plasmid containing the TTR promoter followed by a short multiple cloning site (MCS), but no gene to be expressed (no ORF). This allows any gene to be inserted after the TTR promoter. The backbone plasmid *pCCLsin.cPPT.hPGK.GFP.WPRE* contains unique XhoI and SalI restriction sites which flank the PGK promoter and the GFP gene. Using the above mentioned strategy, the two plasmids (*pCCLsin.cPPT.hPGK.GFP.WPRE*  and *pBS-TTR)* were simultaneously digested with XhoI and SalI enzymes to release the transfer vector and the TTR promoter sequence, respectively. After agarose gel separation and column purification, the two fragments were ligated to create the intermediate TTR transfer vector *pCCLsin.cPPT.hTTR.noORF.WPRE*.

**Plasmid *pCCLsin.cPPT.hTTR.GFP.WPRE*.**

The plasmid *pCCLsin.cPPT.hTTR.noORF.WPRE* provides 3 unique restriction sites after the TTR promoter (SmaI, EcoRV and SalI). In plasmid *pCCLsin.cPPT.hEF1.GFP.WPRE* the target sites for enzymes SmaI and SalI surround the GFP gene. Both plasmids were simultaneously digested with SmaI and SalI; the fragments were gel separated and column purified. *pCCLsin.cPPT.hTTR.noORF.WPRE* and the GFP gene were then ligated to create the transfer plasmid expressing GFP under the TTR promoter *pCCLsin.cPPT.hTTR.GFP.WPRE* (Supplementary Fig. 3).

**Plasmid *pCCLsin.cPPT.EF1.hYap.ERT2.WPRE*.**

After enzyme digestion with SmaI and SalI, the GFP gene sequence was removed from plasmid *pCCLsin.cPPT.hEF1.GFP.WPRE,* which allowed the hYap gene to be inserted. The ORF for the hYap from plasmid P2xFlag CMV2-hYAP1-2β was PCR amplified, using a reverse primer lacking the stop codon. The PCR fragment was digested with SalI and cloned into the aforementioned vector (sticky and blunt end ligation). The final plasmid contained the EF1 promoter followed by the hYap ORF (without a stop codon): *pCCLsin.cPPT.hEF1.hYap.noStop.WPRE.* The ERT2 sequence was PCR amplified from plasmid pCAG-CreERT2 using ER-F and R primers, containing SalI target sites. The design of the forward primer allows the ERT2 sequence to be attached to the Yap gene in frame, so that both peptides will be synthesized as a single molecule. *pCCLsin.cPPT.hEF1.hYap.noStop.WPRE* and the amplified ERT2 fragment were digested with SalI, column purified and ligated together to form the transfer plasmid *pCCLsin.cPPT.EF1.hYap.ERT2.WPRE* (Supplementary Fig. 3).

**Plasmid *pCCLsin.cPPT.TTR.hYap.ERT2.WPRE.***

The plasmid *pCCLsin.cPPT.TTR.noORF.WPRE* provides 3 unique restriction sites after the TTR promoter (SmaI, EcoRV and SalI). To prepare the transfer vector, this plasmid was digested with SmaI and purified. Using Yap-F and ER-R primers, a DNA fragment containing the Yap gene linked to ERT2 was amplified (2513bp). Plasmid *pCCLsin.cPPT.EF1.hYap.ERT2.WPRE* was used as a template and the fragment was gel-purified. Both the blunt end vector and the PCR fragment were ligated to prepare the transfer vector plasmid *pCCLsin.cPPT.TTR.hYap.ERT2.WPRE* (Supplementary Fig. 3).

**Virus production and titration.**

A third generation expression system was used to generate lentiviruses by transient transfection of HEK293T cells, using CaPO_4_ transfection, see Supplementary Reference (1). Four plasmids were provided by the Gene Therapy Core at the Albert Einstein College of Medicine: pMDLg/pRRE (packaging plasmid containing Gag and Pol), pCMV-VSV-G (envelope plasmid), pRSV-Rev and the self-inactivating (SIN) transfer vector plasmids (prepared as indicated above). The calcium phosphate-DNA precipitate was allowed to remain in contact with the cells for 14-16 h, followed by medium replacement. Cell medium was collected 48 h later, centrifuged at 20,000 rpm for 90 min at room temperature and the pellet (viral particles) was resuspended in DMEM medium (1/200 of the initial volume). The virus particle (VP) concentration was determined by qRT PCR, using the Lenti-X RT-PCR titration kit (Clontech Mountain View, CA), according to the manufacturer’s instructions.

**Supplementary Table 1. Primer Sequences used forRT-PCR or qRT-PCR:**

**A. RT-PCR primers**

| **Gene** | **Primer Sequence** | **Sequence Detected** |
| --- | --- | --- |
| Birc2 (cIAPI) | F 5’-AGCTTGCAAGTGCTGGATTT-3’  R 5’-CACCAGGCTCCTACTGAAGC-3’ | 359bp |
| Birc3 (cIAP2) | F 5’-CTAGCCCTCAGCCTCCTCTT-3’  R 5’-GCAAAGCAGGCCACTCTATC-3’ | 281bp |
| Bcl-XL | F-ACCGGAGAGCATTCAGTGAT-3’  R-GCAGAACTACACCAGCCACA-3’ | 374bp |
| Birc4 (XIAP) | F 5’-GCAGTCCTGTTTCAGCATCA-3’  R5’-GGGTTCCTCGGGTATATGGT-3’ | 357bp |
| Birc5 (survivin) | F 5’-TAAGCCACTTGTCCCAGCTT-3’  R5’-TCCATTACCCCATGGTAGGA-3’ | 381bp |
| cFLAR (c-Flip) | F5-CATTCACCAGGTGGAGGAGT-3’  R5’-CGGCCTGTGTAATCCTTTGT-3’ | 346bp |
| GAPDH | F5’-ATCCACTGGTGCTGCCAAG-3’  R5’- ATGTAGGCCATGAGGTCCAC-3’ | 371bp |

**B. qRT-PCR primers**

| **Gene** | **Primer Sequence** | **Sequence Detected** |
| --- | --- | --- |
| qCtgf | F 5’- AGACCTGTGCCTGCCATTAC-3’  R 5’- GCTTTACGCCATGTCTCCAT-3’ | 92bp |
| qGapdh | F 5’-GGCATTGCTCTCAATGACAA-3’  R 5’-ATGTAGGCCATGAGGTCCAC-3’ | 95 bp |

**Supplementary Table 2. Antibodies used to detect specific proteins in repopulating clusters of lenti TTR-Yap-ERT2-transduced DPPIV^+^ hepatocytes transplanted into DPPIV^-^ rat recipient liver**

Primary antibodies

| **Antibodies** | **Isotype** | **Company or Producer** | **Cat. Number** | **Dilution** |
| --- | --- | --- | --- | --- |
| Mouse anti-rat CD26/DPP4 | IgG1 | Santa Cruz Biotech. | sc-52642 | 1:50 |
| Rabbit anti-human albumin | Polyclonal | Gift, Dr. N. Roy-Chowdhury |  | 1:100 |
| Rabbit anti-human HNF-4a | Polyclonal | Santa Cruz Biotech | sc-8987 | 1:50 |
| Rabbit anti-rat ASGPR | Polyclonal | Gift, Dr. R. Stockert |  | 1:100 |
| Rabbit anti-human Yap1 | Polyclonal | Novus Biologicals | NB110-58358 | 1:50 |
| Mouse anti-human Ki-67 | IgG1 | BD Pharmingen | 550609 | 1:50 |
| Mouse anti-human CK19 | IgG1 | Novocastra Laboratories Ltd. | NCL-CK19 | 1:100 |
| Rabbit anti-Sox9 | Polyclonal | EMD Millipore | AB5535 | 1:50 |
| Mouse anti-rat EpCAM | IgG1 | BioVendor, LLC | RD-680 | 1:75 |
| Rabbit anti-human Afp | Polyclonal | Thermo Fisher Scientific | RB-365-A1 | 1:100 |
| Mouse anti-rat OV6 | IgG1 | Gift, Dr. S. Sell |  | 1:50 |
| Rabbit anti-CD133 | Polyclonal | ABCAM | ab19898 | 1:25 |
| Mouse anti-rat CD44 | IgG2a | Cedarlane | CL044 | 1:50 |
| Mouse anti-rat  CD26/DPP4 | IgG2a | Cedarlane | CL061AP | 1:50 |

Secondary fluorescent antibodies

| **Antibodies** | **Fluorofore** | **Company or Producer** | **Cat. Number** | **Dilution** |
| --- | --- | --- | --- | --- |
| Donkey anti-mouse IgG | DyLight 549 | Jackson Immunoresearch | # 715-505-151 | 1:400 |
| Goat anti-mouse IgG1 | cy3 conjugated | Same | #715-165-151 | 1:400 |
| Goat anti-mouse IgG2a | cy3 conjugated | Same | #115-165-206 | 1:400 |
| Goat anti-mouse IgG | cy2 conjugated | Same | #115-225-205 | 1:400 |
| Donkey anti-rabbit IgG | DyLight 488 | Same | #715-095-151 | 1:200 |
| Donkey anti-rabbit IgG | Cy3 conjugated | Same | #711-167-003 | 1:200 |

**Supplementary Reference**

1. Naldini *L, et a*l. (1996) In vivo gene delivery and stable transduction of nondividing cells by a lentiviral vector*. Scien*ce 272(5259):263-267.
